# Mechanisms Underlying the Transmission of Insect Pathogens

**DOI:** 10.3390/insects10070194

**Published:** 2019-07-02

**Authors:** Monique M. van Oers, Jørgen Eilenberg

**Affiliations:** 1 Laboratory of Virology, Wageningen University and Research, Droevendaalsesteeg 1, 6708 PB Wageningen, The Netherlands; 2 University of Copenhagen, Plant and Environmental Sciences, Thorvaldsensvej 40, 1871 Frederiksberg C., Denmark

**Keywords:** entomopathogen, virus, fungus, bacteria, nematode, transmission, virulence, endosymbiont, sporulation, virus entry, infective juvenile, toxin

## Abstract

In this special issue the focus is on the factors and (molecular) mechanisms that determine the transmission efficiency of a variety of insect pathogens in a number of insect hosts. In this editorial, we summarize the main findings of the twelve papers in this special issue and conclude that much more needs to be learned for an in-depth understanding of pathogen transmission in field and cultured insect populations. Analyses of mutual interactions between pathogens or between endosymbionts and pathogens, aspects rather under-represented in the scientific literature, are described in a number of contributions to this special issue.

## 1. Introduction

Insect pathogens include a huge diversity of species. In their text book, Vega and Kaya [1] describe the taxonomy, diversity, and infection biology of a large variety of these pathogens, encompassing different types of insect viruses (DNA and RNA viruses), the main insect pathogenic fungal orders (Hypocreales, Ascosphaerales, and Entomophthorales), and important insect bacterial groups (gram-positive genera like *Bacillus* and gram-negative genera like *Serratia*, as well as genera of endosymbionts like *Wolbachia*). Furthermore, microsporidian insect pathogens (Gregarines), as well as nematodes (genera *Steinernema* and *Heterorhabditis*) acting with their associated bacterial symbionts, were included. Clearly, such a diverse collection of (micro-)organisms will exhibit a large variation in their transmission strategies and associated mechanisms. The transmission of an insect pathogen from a diseased, infectious host to uninfected, susceptible insects requires a combination of pathogen characteristics, host properties, and environmental biotic and abiotic factors [2]. For this special issue of *Insects*, we have collected a set of twelve articles that reflect this diversity in transmission strategies. The articles differ with respect to pathogens and hosts, and vary in scientific focus, as indicated below.

## 2. Content Overview

Several papers deal with the transmission strategies of fungi. The article by Angelone et al. [3] deals with the genus *Metarhizium* and suggests that for this group of insect pathogenic fungi polymorphism with respect to transmission in soil exists. One strategy is to be what they call a “sleeper”. This means that the fungus phenotype produces high amounts of conidia, which remain in the soil and wait until they are picked up by a suitable host to start infection. The other strategy, being a “creeper”, means that this phenotype will produce less conidia, but more mycelia, allowing hyphae to grow through the soil and increase the chance of encountering a host. The soil as a reservoir for the prolonged survival of resting spores of insect pathogenic fungi from the order Entomophthorales and the horizontal transmission of these fungi is the topic of the article by Hajek et al [4]. In order to infect, these resting spores must produce an infective conidium. These authors compare different host–fungal pathogen systems (amongst others, the gypsy moth *Lymantria dispar* with the fungus *Entomophaga maimaiga* and periodical cicadas in combination with *Massospora* species). In the study by Castro et al. [5], the role of secondary conidia with respect to transmission of the fungus *Neozygites floridana* to its host, the spider mite *Tetranychus urticae,* is explored by determining the environmental conditions that enhance the chances for transmission. Haelewaters and co-authors [6] describe natural infections of the invasive harlequin ladybirds (*Harmonia axyridis*) with a fungus *Hesperomyces virescens* from the order Laboulbeniales, and found that while the size of the ladybird influenced the prevalence of the fungus, the color patterns as expressed by the melanization of the ladybird’s elytra did not.

The entry mechanisms of several types of pathogens are described in a number of papers. The contribution by Boogaard et al. [7] provides an overview of the current knowledge on baculovirus *per os* infectivity factors (PIFs), which are crucial for the horizontal transmission of baculoviruses through oral infection. Most of these PIF proteins assemble into a multimeric complex. The existence of this complex makes it difficult to determine the role of the individual PIF proteins in the midgut infection process. The article by Buisson et al. [8] discusses whether *Bacillus thuringiensis* toxin crystals, encoded by the bacterial PlcR virulence regulon and exploited by the bacteria PlcR virulence regulon and during oral infection, can also assist in infecting the haemocoel upon cuticle damage. The authors show that the bacterial spores are more virulent than vegetative *B. thuringiensis* cells when injected into *Galleria mellonella* larvae, and that spores that lack the toxins are less virulent than wild type spores. The article by Maciel-Vergara et al. [9] presents a study about the infection routes of the opportunistic bacteria of the genus *Pseudomonas* and documents an infection route for larvae of the giant mealworm, *ZophobasZophobas morio*, via wounds, which appears to be important in insect production systems. They also show that cannibalism and scavenging are more prevalent among larvae infected with *P. aeruginosa*.

The paper by Alonso et al. [10] aims at unravelling factors important for the activation of infective juvenile nematodes. This activation is the first crucial step in parasite infestation and involves the release of a bacterial symbiont, secretion of toxic products, and morphological adaptations of the nematode. These authors show that, for *Steinernema carpocapsae* and *S. feltiae*, the host tissue and the age of the infective juveniles are important determinants for activation and hence for virulence. The paper by Labaude and Griffin [11] reviews the transmission success of the generalist insect pathogens from the nematode genera *Steinernema* and *Heterorhabditis* and outlines the many biotic and abiotic factors involved. In addition, these authors list novel species in these genera that may prove very interesting to study. Nematodes from the above-mentioned genera work in joint action with gram-negative bacteria from the genera *Photorhabdus* and *Xenorhabdus.* These bacteria (and other gram-negative insect pathogenic bacteria) are the main subject of the article by McQuade and Stock [12]. They highlight how secreted proteins may play different roles in pathogenesis. They also indicate that non-protein metabolites released by these bacteria can prove to have potential as novel biopesticides. 

Two other manuscripts also deal with interactions between micro-organisms. The paper by Sinotte et al. [13] focuses on mutualisms between ants of the genus *Camponotus* and endosymbiotic bacteria (*Blochmannia*) and elucidates how this interaction affects ant development and resistance against fungi. Interestingly, they found that when infected with the symbiotic bacterium *B*. *floridanus*, *C. floridana* ants had a decreased level of melanization and, as a consequence, were more susceptible to infections by the fungus *Metarhizium brunneum*. In the study by Pauli et al. [14], simultaneous infections with two fungi or with a fungus and a granulovirus (family *Baculoviridae*) were analyzed. The combination of the fungus *Beauveria bassiana* and the virus resulted in a certain level of antagonism, while *Metarhizium anisopliae* showed an additive effect to viral infection of the sugarcane borer *Diatraea saccharalis*. There was clear competition between the viral and fungal infections, as finally only one of the two was amplified to transmittable amounts. When co-inoculation was performed with the fungal species, both were able to efficiently produce spores, indicating that they did not outcompete each other.

## 3. Conclusions

From the diverse set of articles in this special issue, it clearly appears that the mechanisms underlying transmission of insect pathogens is a research area of high interest. Basic biological features about hosts and pathogens need to be unraveled for an in-depth understanding of the transmission processes. Each article in this special issue has recommendations for future studies, and we can conclude that there are many exiting discoveries to be made. We especially recommend further efforts to shed light on the interactions between two types of insect pathogens simultaneously present in a single host. There is also more to discover in understanding the specific roles of different toxins and other proteins in the initial infection processes, and how these proteins interact with each other and the host for optimal functionality. From the applied side, it is evident that the better the understanding that we have obtained and the better the biological control products that we can develop, the better we will be able to control pest insect populations, for instance in agricultural settings. Gathering additional information on interactions between insect pathogens is also of evident importance for natural settings, given the current decline of insect populations worldwide, as well as for preventing disease outbreaks in the upcoming industry of insect rearing for a variety of applications. 
